# The cure from within? a review of the microbiome and diet in melanoma

**DOI:** 10.1007/s10555-022-10029-3

**Published:** 2022-04-27

**Authors:** Priyanka Kumar, Danielle Brazel, Julia DeRogatis, Jennifer B. Goldstein Valerin, Katrine Whiteson, Warren A. Chow, Roberto Tinoco, Justin T. Moyers

**Affiliations:** 1 Department of Medicine, University of California Irvine, Orange, CA USA; 2 Department of Molecular Biology and Biochemistry, School of Biological Sciences, University of California, Irvine, CA USA; 3 Division of Hematology and Oncology, Department of Medicine, University of California Irvine, 101 The City Drive South, Building 200, Orange, CA 92868 USA

**Keywords:** Microbiome, Immunotherapy, Checkpoint inhibitors, Metastatic melanoma, Cutaneous melanoma

## Abstract

Therapy for cutaneous melanoma, the deadliest of the skin cancers, is inextricably linked to the immune system. Once thought impossible, cures for metastatic melanoma with immune checkpoint inhibitors have been developed within the last decade and now occur regularly in the clinic. Unfortunately, half of tumors do not respond to checkpoint inhibitors and efforts to further exploit the immune system are needed. Tantalizing associations with immune health and gut microbiome composition suggest we can improve the success rate of immunotherapy. The gut contains over half of the immune cells in our bodies and increasingly, evidence is linking the immune system within our gut to melanoma development and treatment. In this review, we discuss the importance the skin and gut microbiome may play in the development of melanoma. We examine the differences in the microbial populations which inhabit the gut of those who develop melanoma and subsequently respond to immunotherapeutics. We discuss the role of dietary intake on the development and treatment of melanoma. And finally, we review the landscape of published and registered clinical trials therapeutically targeting the microbiome in melanoma through dietary supplements, fecal microbiota transplant, and microbial supplementation.

## Introduction

The melanoma mortality rate was stable between 1989 and 2013; however, a dramatic decline in mortality of − 5.7% per annum occurred between 2013 and 2018 [1, 2]. Melanoma remains the deadliest of the skin cancers with 7650 deaths and 99,780 new diagnoses expected in the USA in 2022 [3]. This shift was created by the shift from less effective biochemotherapies including high dose interleukin-2 and interferon-alfa to a second era of immunotherapy with checkpoint inhibitors [4, 5].

Improved overall survival (OS) with Ipilimumab, an anti-cytotoxic T-lymphocyte-associated antigen 4 (CTLA-4) agent, was seen [6, 7]. Subsequently, pembrolizumab and nivolumab, both anti-program death 1 (PD-1) antibodies, were shown to have remarkable efficacy alone and for nivolumab in combination with ipilimumab [8–10]. With additional checkpoint therapies including relatlimab, an anti-LAG3 antibody (lymphocyte-Activation Gene 3) and other promising combinations are on the horizon [11–13].

While variation between groups of people has often been explained through genetic variation of human genes, the differences in the composition of microbes within our body represent another source of human diversity [14]. Recent high-profile works have catapulted the microbiome into the spotlight; however, will it usher in the third era of immunotherapy for the treatment of melanoma? In this review, we discuss the role of the microbiome in the development melanoma. We then discuss the microbiome’s potential role in predicting immunotherapy response before exploring its therapeutic potential.

## Melanoma and the Skin microbiome

Millions of bacteria, viruses, and fungi call our skin home and compose the microbiota of the skin. They play important roles in protecting against pathogens and educating our immune system [15, 16]. The composition of the skin microbiota varies by site (e.g., feet versus head) and skin type (e.g., cutaneous versus mucosal) and may become dysbiotic in disease states [17]. While ultraviolet (UV) exposure and genetic predispositions are most associated with tumorigenesis, much is unknown on the role the skin microbiota has in this processes [18].

Several viruses have been linked to the development of other cancers including serotypes of human papilloma viruses in cervical and head and neck cancers and polyomavirus in Merkel cell carcinoma, a neuroendocrine tumor of the skin, although a causative viral etiology has not been identified in cutaneous melanoma [19, 20]. Bacterial infection with a Marjolin’s ulcer has been associated with cutaneous squamous cell carcinoma; however, no bacterial skin infections have yet been identified as causal in melanomagenesis [21].

Small studies have shown that there is a difference in the microbiota of skin with melanomas. A study of 15 cutaneous melanomas and 17 benign melanocytic nevi characterized the microbiome of skin samples via 16 s RNA gene sequencing. *Cutibacterium acnes* (formerly Propionobacterium) was the most common genus along with *Staphylococcus* spp.and *Corynebacterium* spp.; however, no signficiant differences in the relative compositional makeup were noted but were noted to have decreased diversity [22]. A separate study retrospectively analyzing 27 bacterial cultures from acral melanoma patients in Japan found corynebacterium to be more common in advanced (stage III/IV) versus early stage acral melanoma (I/II) [23].

The microbiota of the skin may harbor intrinsic anti-cancer protective effects. Cis-Urocanic acid is an endogenous compound of the skin; in models, it is able to inhibit melanoma growth via the acidifcation of the cytosol of tumor and stromal cells [24]. *Staphylococcous epidermidis*, a common skin commensal microbe, when restored in germ-free mice has been shown to normalize IL-17A production, a chemokine which may a play a role in tumor growth and anti-tumor immunity [25, 26]. An additional strain of *S. epidermidis* which produces 6-N-hydroxyaminopurine (6-HAP), an inhibitor of DNA polymerase activity, can suppress melanoma B16F10 growth. Mice colonized with with 6-HAP producing *S. epidermidis* had reduction in incidence of UV-induced skin tumors compared to controls [27].

For the purposes of this review, we did not consider oncolytic viruses as microbiome altering therapies. Oncolytic viruses are genetically modified to enhance tumor tropism to stimulate a proinflammatory environment to active the immune system [28]. However, it should be noted the modified herpes simplex virus type 1-derived oncolytic immunotherapy talimogene laherparepvec (T-VEC) as intratumoral injection is FDA approved in melanoma [29]. Many more trials are ongoing utilizing T-VEC and similar oncolytic viruses alone (e.g., NCT03989895, NCT04427306) and in combination with checkpoint inhibitors (e.g., NCT05070221, NCT04370587, NCT04570332, NCT04695977, NCT04348916).

Ventures are ongoing to harness the therapeutic, prognostic, and predictive potential of the gut microbiome, but such an effort has yet to take place for the skin microbiome. Given the potential role in cancer development and changes in a disease state, it may be the next frontier in melanoma microbiome research [30]. And while interventional trials are lacking, the newly registered SKINBIOTA trial (NCT04734704) will examine the skin microbiome from swabs of 175 melanoma patients on anti-PD-1 therapy as standard of care.

## Microbiome and the immune system

**Fig. 1:**
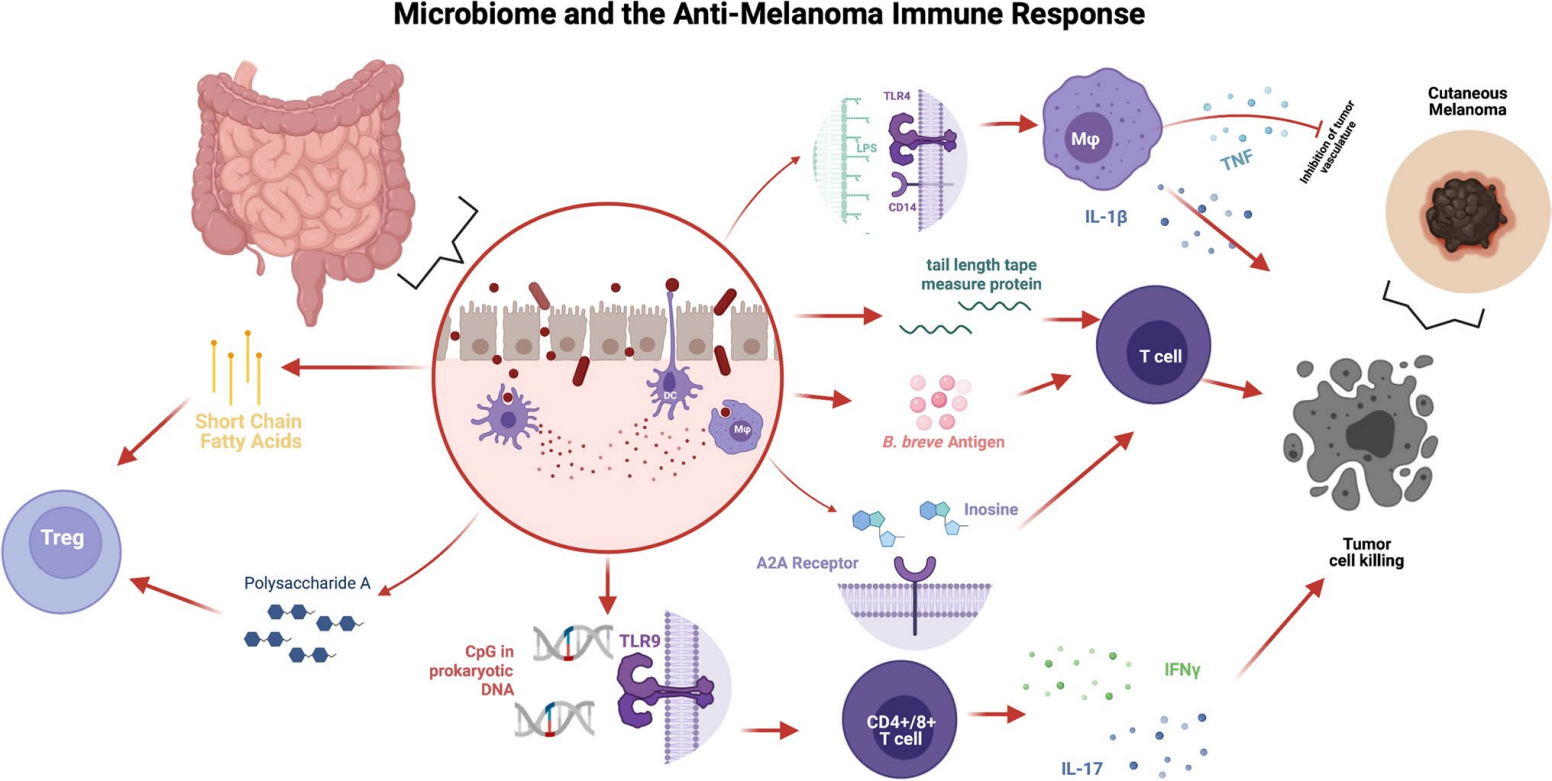
Interplay between cutaneous melanoma and the microbiome: At the level of the intestinal wall (in circle) short chain fatty acids and polysaccharide A from bacteria induce a regulatory environment through activation of T regulatory cells. CpG DNA motifs common in prokaryotic DNA include a response from TLR9 on CD4 + /8 + T cells to produce inflammatory cytokines IL-17 and interferon gamma. Tail length tape measure protein and inosine produced in bacteria in the gt also promote T-cells which can lead to enhanced anti-tumor response. Furthermore, lipopolysaccharides (LPS) on the cell membranes of bacteria are recognized by TLR4 on macrophage and other dendritic cells causing release of IL-1Beta and TNF. Created with biorender.com

The microbes inhabiting the gut include bacteria, fungi, protozoa, viruses and bacteriophages. It is estimated that up to 4 × 10^13^ microbial cells, mostly bacteria, are present in the human body with greater than 95% of these living in the gut [31, 32]. Given the high abundance of microbes and the large size of this organ system, the gut represents a major player in the regulation of immune responses in cancer. It has become increasingly clear that patient responses and treatment outcomes are influenced by gut health and dysbiosis, and that the microbiome and immunotherapeutic response are in fact intrinsically tied [33]. Bacterial fermentation products, such as short chain fatty acids (SCFAs), maintain immunosuppressive regulatory T cells (Tregs) in the intestine, and can also downregulate histone deacetylases (HDACs), leading to hyperacetylation of histones in immune cells and resultant downregulation of pro-inflammatory cytokines [33, 34]. Additionally, polysaccharide A (PSA), which is made by symbiotic *Bacteroides fragilis* in the gut, can expand IL-10-producing Tregs and shift gut immunity toward a more immunosuppressive phenotype [35]. The gut microbiome can also promote inflammatory responses. Unmethylated cytosine phosphate guanosine (CpG) dinucleotides, present in high levels in prokaryotic DNA, are recognized by TLR9 and are important in promoting inflammatory IFN-γ producing CD4^+^ and CD8^+^ T cells, as well as IL-17 production in CD4^+^ T cells [36]. The importance of the gut microbiome on immune homeostasis has been demonstrated in germ-free (GF) mice, which showed an increase in circulating mast cells, reduced phagocytic function in neutrophils and macrophages, and decreased numbers of gut DCs [37]. Preclinical and clinical data has demonstrated an important regulatory function by the gut microbiota in oncogenesis, progression, and response to immunotherapy [38]. Figure 1 summarizes select key pathways in this complex system.

### Preclinical models of the gut microbiome and cancer immunotherapy

Preclinical murine models have been an important strategy for understanding microbiome and immune system interactions in melanoma immunotherapy. Sivan et al. found tumor-bearing mice that were separately housed had distinct microbiota and significantly different anti-tumor immune responses. This effect was abrogated if mice were co-housed. Anti-PD-1 immune checkpoint inhibitor (ICI) therapy treatment resulted in even more stratified anti-tumor immunity between separately housed mice, with one set achieving increased effector T cell functions and melanoma tumor control. *Bifidobacterium* was found to be associated with improved anti-PD-1 responses and therapeutic administration of *Bifidobacterium*-enhanced anti-PD-1 ICI response in previously non-responding mice [39]. Much is unknown about the mechanisms through which bacteria promote anti-tumor immunity; however, T cells specific for microbial antigens in mice were shown to be cross-reactive to melanoma antigens. T cell specificity for tail length tape measure protein (TMP) from *Enterococcus hirae* bacteriophage and T cells specific for *Bifidobacterium breve* antigen was shown to cross-react to melanoma tumor cells [40, 41]. Furthermore, anti-PD-1 therapy was improved in the presence of TMP-containing enterococci.

In a separate study, the microbiome was shown to drive anti-tumor responses to CTLA-4 blockade in mice. Vetizou et al. showed enrichment of *Bacteroides* spp. in the microbiome of tumor-bearing mice responsive to anti-CTLA-4 therapy. Additionally, tumors progressed in antibiotic-treated or germ-free mice (GF) during anti-CTLA-4 therapy, showing the importance of the microbiome in the anti-CTLA-4 response [42]. Germ-free tumor bearing mice fed oral *Bacteroides* spp. and anti-CTLA-4 showed anti-tumor immune response with increased T_H_1, DC maturation, and improved tumor control. To increase the translational relevance of these findings, the gut microbiomes of metastatic melanoma patients treated with ipilimumab were analyzed and found to have had increased levels of *Bacteroides salyersiae*, *Bacteroides acidfaciens*, *Bacteroides uniformis*, and decreased levels of *Prevotella copri*, *Bacteroides sp*., *Barnesiella intestinihominis*, and *Parabacteroides distasonis* after treatment [42]. Fecal microbial transplantation (FMT) studies from metastatic melanoma patients into GF tumor-bearing mice found that increased abundance of *Bacteroides* spp. correlated with smaller tumor sizes, whereas reconstitution with *Bacteroides fragilis* and *Burkholderia cepacia* reduced colitis like histopathological changes from anti-CTLA-4, hypothesizing that *Bacteroides fragilis* elicits an IL-12-dependent T_H_1 immune response.

To combine clinical data with pre-clinical studies, Matson et al. administered FMT from metastatic melanoma ICI responders or non-responders to germ-free melanoma-tumor bearing mice [43]. The mice that received responder fecal material showed significantly improved melanoma tumor control both with and without anti-PD-1 treatment. Further mechanistic studies revealed that the recipients of responder fecal material had increased numbers of tumor-specific CD8^+^ T cells and IFN-γ production. Mice receiving FMT from non-responding patients did not achieve any benefit from anti-PD-1 treatment, demonstrating the pre-clinical impact that microbiome can have on ICI efficacy. In a similar approach, Gopalakrishnan et al. performed FMT from anti-PD-1 responsive and non-responsive patients into germ free mice, which were then injected with melanoma cells and treated with anti-PD-L1 [44]. Mice with microbiota derived from responders showed significantly increased melanoma tumor control, as well as increased CD8^+^ T cell density in the tumor. While FMT studies have shown promise in melanoma immunotherapy, this strategy can also introduce potentially fatal pathogens [45].

From an isolate of 11 bacterial strains obtained from healthy human fecal donors, several permutations of bacterial mixtures were optimized for testing. An 11-mix containing 3 *Parabcteroides* species, one *Alistripe paraprevotella*, *Bacteroides dorei*, *Bacteroides uniformis*, *Eubacterium limosum*, *Ruminocaccaceae bacterium*, *Phascolarctobacterium faecium*, and *Fusobacterium ulcerans* was found to induce the best response. This mixture was capable of inducing a robust interferon-gamma-producing CD8 T-cell response  and improve the therapeutic efficacy of checkpoint inhibitors in syngeneic tumor models [46], supporting the potential for a “designer mix” therapy.

Mager et al. cultured intratumoral microbes and found that colonizing germ-free mice with *Bifidobacterium pseudolongum*, *Lactobacillus johnsonii*, or *Olsenella* promoted anti-CTLA-4 responses and tumor control [47]. The administration of these microbes also increased IFN-γ production in intratumoral T cells. Further investigation found that the metabolite inosine, which is produced by *B. pseudolongum* as well as another ICI response-promoting bacteria *Akkermansia muciniphila*, promotes anti-tumor immunity and improved ICI efficacy in mice. This effect was dependent on expression of adenosine A_2A_ receptor, an immune negative feedback mechanism, and T cell co-stimulation representing an important step toward understanding the direct mechanisms through which bacteria impact immune responses to ICI [48].

### Checkpoint inhibitors and microbiome in melanoma

Melanoma is one of the most highly mutated cancers, while these mutations facilitate resistance to chemotherapies and targeted therapy, and the high mutational burden also leads to the generation of neoantigens recognized by the immune system [49]. Checkpoints are inhibitors of anti-tumor T cells; CTLA-4 prevents T cell activation while PD-1 functionally inactivates TCR and CD28 signaling, dampening T cell effector function [50, 51]. Antigen-specific CD4^+^ and CD8^+^ T cells in tumors are functionally exhausted and can be reinvigorated using immune checkpoint inhibitors which result in tumor destruction; however, efficient presentation of tumor antigens appears to be key to ICI efficacy [52]. Development of the innate immune system is affected by the microbiome with distinct effects homeostasis, myeloid maturation, and antigen presentation [53]. ICIs are now the standard first-line therapy for metastatic melanoma, and ICIs given in combination with one another or along with other anti-cancer modalities have the potential to increase efficacy or overcome resistance [12, 13, 54–57]. However, efficacy of immune activation by checkpoints may be increased identifying the characteristics of the “optimal microbiome” and its effects on antitumor immune response, antigen presentation, and effector T-cell function in the periphery and tumor microenvironment [44].

### Commensal bacteria as checkpoint inhibitor biomarkers; friends and foes:

In the first prospective study reported, Frankel et al. collected baseline fecal samples of 39 patients with unresectable or metastatic melanoma prior to treatment with ipilimumab, nivolumab, ipilimumab plus nivolumab, or pembrolizumab. Samples from responders to nivolumab and ipilimumab plus nivolumab were enriched with *Faecalibacterium prausnitzii*, *Holdemania filiformis*, and *Bacteroides thetaiotamicro*n. Responders treated with pembrolizumab had higher baseline levels of *Dorea formicigenerans*. The authors did not find an association between microbial diversity and response to treatment [58].

A similar study performed by Wind et al. profiled the gut microbiome of 25 patients (12 responders) utilizing metagenomic shotgun sequencing of pre-treatment stool samples. In this cohort, no significant differences in alpha-diversity (diversity of mean bacteria within a site, e.g., patient) or bacterial prevalence were detected between responders and non-responders; however, analysis of 68 bacterial taxa did show differences [59]. Prolonged overall survival (OS) or progression-free survival (PFS) was seen in carriers of *Streptococcus parasanguinis* or *Bacteroides massiliensis*, respectively, while shorter OS and PFS was seem in Peptostreptococcaceae carriers.

Chatput et al. used 16S rRNA gene sequencing to examine the microbiota of 26 metastatic melanoma patients before, during, and after four cycles of ipilimumab. Patients were then categorized into baseline microbiota drivers (cluster A driven by *Faecalibacterium* and other Firmicutes, cluster B driven by *Bacteroides*, and cluster C driven by *Prevotella*). Patients with baseline microbiomes that fell into cluster A showed statistically significant longer PFS, OS, and had lower baseline levels of Tregulatory cells. Responders had a higher proportion of *Faecalibacterium, Clostridium*, and *Gemminger.* Additionally, higher levels of *Ruminococcus* and L*achnospiraceae* at baseline were associated with an overall survival of greater than 18 months. Non-responders had higher levels *Bacteroides* (*p* = 0.034). These findings were independent of antibiotic use [60].

Matson et al. evaluated the gut microbiota composition of 42 metastatic melanoma (16 responders, 26 non-responders) utilizing 16*S* rRNA gene amplicon sequencing before and after anti-PD-1 (*n* = 38) or anti-CTLA-4 (*n* = 4) [43]. Analysis of sequencing results revealed that the family of *Bifidobacteriaceae* was significantly more abundant in responders than non-responders. Additional species that were enriched in the responder group included *Enterococcus faecium*, *Collinsella aerofaciens*, *Bifidobacterium adolescentis*, *Klebsiella pneumoniae*, *Veillonella parvula*, *Parabacteroides merdae*, *Lactobacillus species*, and *Bifidobacterium longum*. The species more abundant in the non-responder cohort included *Ruminococcus obeum* and *Roseburia intestinalis*. Furthermore, a ratio over 1.5 of good (e.g., bacteria associated with clinical response) to bad bacteria stratified responders from non-responders, possibly serving as a new biomarker to predict ICI responses in the clinic.

The fecal and oral microbiota composition from 112 metastatic melanoma patients before and after anti-PD-1 therapy by Gopalakrishnan et al. included 30 responders and 13 non-responders in the fecal microbiome cohort and found higher alpha-diversity in responders (*p* < 0.01) than non-responders [44]. Additionally, higher baseline fecal alpha-diversity correlated with prolonged PFS when compared to intermediate and low alpha-diversity and *Faecalibacterium* genus was found to be enriched in anti-PD-1 responders. Furthermore, *Clostridiales* and *Ruminococcaceae* were enriched in the fecal microbiome of responders and *Bacteroidales* was enriched in non-responders (*p* < 0.01). Analysis of the abundance of *Faecalibacterium* and *Bacteroidales* with respect to disease progression showed that high abundance of *Faecalibacterium* and low abundance of *Bacteroidales* were significantly correlated with higher PFS. Patient samples showed increased CD8^+^ T cell density in responders, and combined gut microbiome and tumor analysis demonstrated that *Faecalibacterium* is associated with increased T cell activation and MHC II upregulation. A recent meta-analysis of microbiome composition in ICI responders compared to non-responders also determined *Faecalibacterium* to be the dominant species in responders [61]. This study also revealed differences in bacterial metabolism between ICI responders and non-responders, with responder samples showing upregulated B-vitamin metabolism pathways, and non-responders showing increased expression of aerobic respiration genes.

The largest trial of microbiome characterization to date was by McCulloch et al. and included 94 PD-1 treated patients with samples collected prior to or within 4 months of start to treatment (*n* = 63) or more than 4 months of treatment (*n* = 31). Comparing the best response as progressors (stable disease < 6 months or progressive disease) and non-progressors (complete or partial response or stable disease ≥ 6 months), Actinobacteria phylum and Lachnospiraceae family were associated with non-progressors while *Bacteroides* and *Proteobacteria* species were associated with progressors [62].

**Fig. 2:**
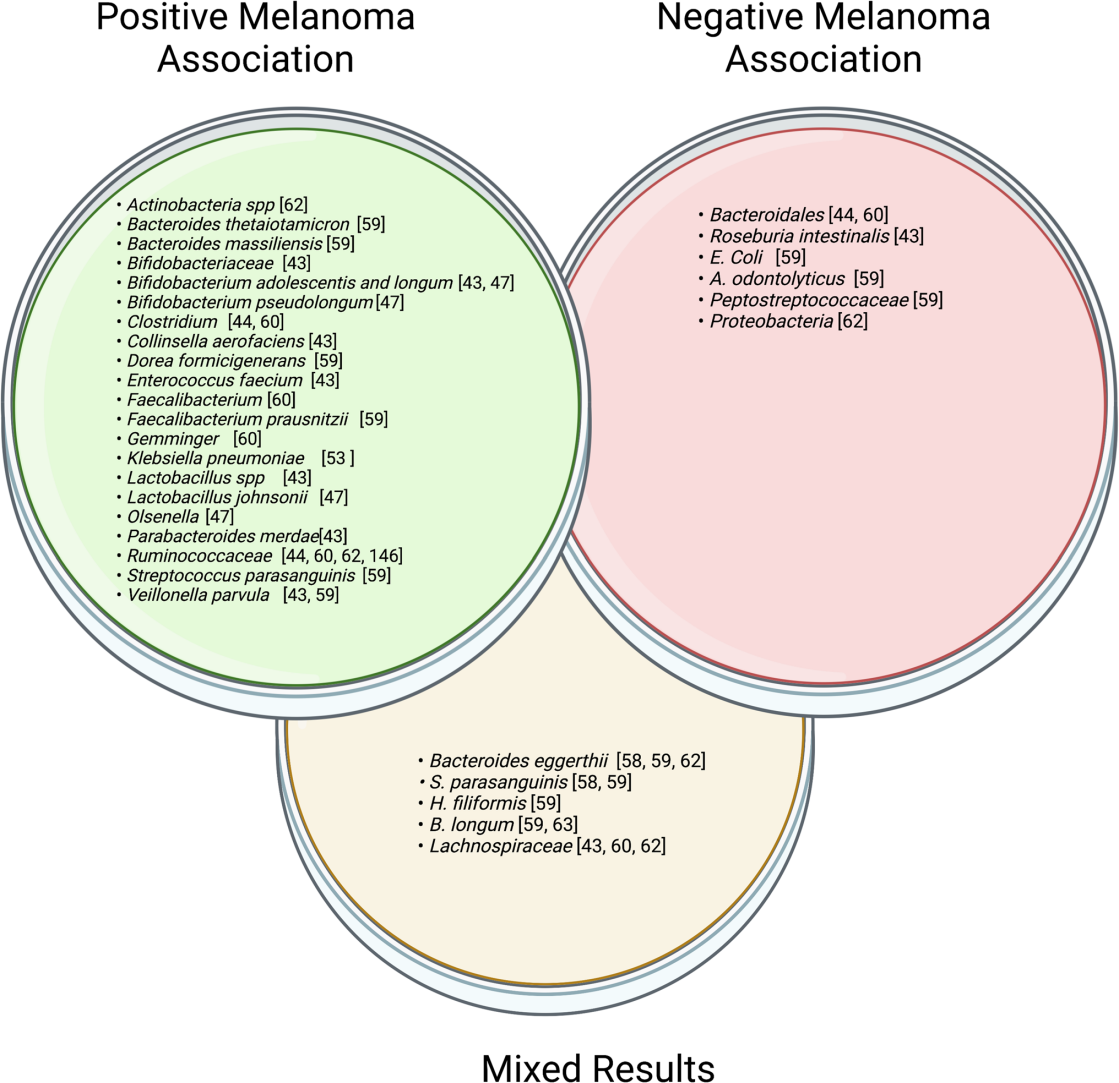
Green petri dish signals gut microbes associated with beneficial effect on melanoma, red petri dish shows gut microbes with negative effect on melanoma, while yellow shows gut microbes with mixed responses reported. Created with biorender.com

Overall, while studies have shown a significant association between certain bacterial taxa and ICI response, these findings remain to be tested and validated in larger prospective clinical trials. The current body of research clearly shows that immune responses are not separate from the microbiome, and that the microbiome should be considered both to predict clinical responses and as an avenue for possible clinical intervention to increase ICI efficacy. Figure 2 summarizes bacteria associated with positive response and those associated with negative response to checkpoint inhibitors.

A list of ongoing registered observational trials examining the interplay of microbiota and checkpoint inhibitors in melanoma can be seen in Table 1. We hope these larger studies will further define the interplay between the microbiome and treatment response to define the bacteria which are markers of a robust immune system and general health versus therapeutic effectors/modulators [63].

**Table 1:** Published and ongoing therapeutic and observational microbiome intervention trials

NCT	Trial phase/type	Patient selection	Treatment	*N*	CBR	ORR	Status	Ref
Procedural FMT								
NCT03341143	Pilot/phase 1	PD-1 refractory	Pembrolizumab + FMT via colonoscopy^1^	16	6/15	3/15	Results published	[153]
NCT03353402	Phase 1	PD-1 refractory	Nivolumab + oral FMT^2^	10	3/9	3/9	Results published	[154]
NCT04577729	Randomized	ICI refractory metastatic melanoma	Checkpoint inhibitor + FMT^4^ versus sham FMT	60*	NR	NR	Recruiting, Medical University Graz	
NCT03819296	Phase 1/2	Checkpoint inhibitor with GI complications in melanoma, lung, and GU	Endoscopic FMT	800	NR	NR	Recruiting, MD Anderson Cancer Center (Houston, TX)	
NCT04988841 (PICASSO)	Phase 2, randomized	Checkpoint naïve unresectable or metastatic melanoma	Checkpoint inhibitor + MaaT013^a^ enema versus placebo	60*	NR	NR	Not yet recruiting, Hôpitaux de Paris	
NCT05251389	Phase 1	Checkpoint refractory melanoma	Endoscopic placed FMT	24	NR	NR	not yet recruiting, The Netherlands Cancer Institute	
NCT05273255	Pilot	Checkpoint refractory melanoma	Endoscopic placed FMT	30	NR	NR	Recruiting, University of Zurich	
Oral FMT/microbial supplement								
NCT04951583 (FMT-LUMINATE)	Phase 2, single group	Untreated NSCLC and melanoma	Nivolumab + Ipilimumab + FMT capsules	70*	NR	NR	Not yet Recruiting, CHUM	
NCT03772899 (MIMic)	Phase 1	Unresectable or metastatic melanoma	FMT^3^ capsule + checkpoint	20*	NR	NR	Active, not recruiting, multiple Canadian sites	
NCT04521075	Phase 1b	Stage IV NSCLC and unresectable and metastatic melanoma	Nivolumab + FMT^4^ oral capsules	42*	NR	NR	Recruiting, Sheba medical center	
NCT03934827 (MICROBIOME)	Phase 1	Resectable select solid tumors (including melanoma)	MRx0518^b^ capsules versus placebo BID 2–4 weeks prior to surgery	120*	NR	NR	Recruiting, Imperial College London	
NCT03817125	Phase 1b	Unresectable or metastatic melanoma	Nivolumab + SER-401^c^ versus placebo	10	NR	NR	Active not recruiting, multiple US sites	[163, 164]
Behavioral diet intervention								
NCT04866810 (EDEN)	Randomized behavioral intervention	Untreated unresectable or metastatic Melanoma	Anti-PD1/PDl1 + observation vs. behavioral diet	60*	NR	NR	Recruiting, National Cancer Institute (Bethesda, MD)	
NCT04645680 (DIET)	Phase 2, randomized	Stage 3 or 4 melanoma	Standard of care immunotherapy with dietary intervention (isocaloric high fiber vs. isocaloric whole foods diet)	42	NYR	NYR	Recruiting, MD Anderson (Houston, Texas USA)	
Observational trials								
NCT	Patient Selection	Treatment	Test/observation	Primary Outcome	N	Status		
NCT04107168 (MITRE)	Stage 3 or 4 melanoma, advanced renal cell carcinoma, advanced NSCLC	Anti-PD-1 or anti-PD-1 with anti-CTLA4	Saliva and stool samples		1800 (up to 360 healthy controls)	Recruiting, multiple sites in UK		
NCT03643289 (PRIMM)	Stage 3 or 4 melanoma naïve to immunotherapy	Standard of care immunotherapy	Gut microbiome with metagenomics of stool samples with diet survey		450	Recruiting, multiple sites in UK		
NCT04734704 (SKINBIOTA)	Melanoma on immunotherapy and non-melanoma vitiligo	Anti-PD-1 as standard of care	Skin swabs on lesional and non-lesional sites		175	Not yet recruiting, Hopital Saint-Andre (Bordeaux, France)		
NCT05037825 (PARADIGM)	NSCLC, Malignant melanoma, RCC, TNBC	Anti-PD-1, anti-PD-L1, anti-CTLA-4 as single agents or in combinations	Longitudinal stool specimens		800	Recruiting, Baptist Health Clinical Research (Elizabethtown, Kentucky, USA)		
NCT03643289 (PRIMM)	Stage 3 and 4 melanoma	Checkpoint inhibitors	Stool sample		450	Recruiting, Multiple Institution, UK		
NCT05102773	Stages 3 and 4 melanoma	Checkpoint inhibitors	Stool and blood samples	Alpha-diversity change	89	Recruiting, Single Institution, Ohio State University, (Columbus, Ohio)		
NCT04875728	Stage I–II melanoma	Surgery + cefazolin surgical prophylaxis	Stool sample	Change of microbiome after prophylactic antibiotics	20	Recruiting, MD Anderson (Houston, Texas USA)		
NCT04136470	Melanoma and NSCLC	Checkpoint inhibitors	Stool sample	Microbial diversity as assessed in gut microbiome	130	Recruiting, Multiple sites (Poland)		
NCT04698161 (BIOMIS-Onco)	Melanoma and NSCLC	Checkpoint inhibitors	Stool, blood, saliva, and urine	Microbe biobank collection	50	IRCCS Istituto Tumori Giovanni Paolo II (Bari, Italy)		
NCT02600143 (COLIPI)	Melanoma with colitis	Checkpoint inhibitors	Stool samples	Longitubindal Gut microbiome differences in colitis development	123	UMCG (Groningen, Netherlands)		

^*^Planned enrollment, **status per clinicaltrials.gov. ^A^MaaT013 is a microbiome restoration biotherapeutic composed of pooled-donor, full ecosystem intestinal microbiome of approximately 455 species. ^B^MRx0518 is lyophilized formulation of a proprietary strain of enterococcus species of bacterium. ^C^SER-401 a purified suspension of firmicute spores from healthy human donors formulated into capsules. *CBR* clinical benefit rate, *CHUM* Centre hospitalier de l'Université de Montréal, *FMT* fecal microbiota transplant, *GU* genitourinary, *NR* not reported, *NSCLC* non-small cell lung cancer, *ORR* objective response rate, *UPMC* University of Pittsburgh Medical Center. ^1^FMT derived from healthy donors who achieved complete response or partial response with PD-1 therapy. ^2^FMT derived from 2 healthy donors who achieved complete response or partial response with PD-1 for > 1 year. ^3^FMT derived from 2 healthy donors per institutional guidelines. ^4^FMT will be given from 1 of 5 donors. Donors will be patients with metastatic melanoma achieving remission for ≥ 1 year

### Antibiotics and the immune response

The careful maintenance of immune homeostasis by a diverse gut microbiome is disrupted by the use of antibiotics, often with negative systemic outcomes [64]. Mice treated with a combination of vancomycin, neomycin, metronidazole, and ampicillin prior to influenza infection showed significantly diminished antigen-specific T cell responses, reduced T cell cytokine production, and increased viral titers [65]. Antibiotic treatment lowered pro-inflammatory IL-1β and IL-18 levels in the lungs and inhibited dendritic cell (DC) migration to mesenteric lymph nodes. A separate study showed that broad-spectrum antibiotic-treated mice had impaired innate and adaptive responses to *Lymphocytic choriomeningitis* virus (LCMV) when compared to untreated mice [66]. Antibiotic treatment also resulted in more weight loss and mortality in influenza-infected mice, as well as impaired CD8^+^ T cell function and anti-viral macrophage responses, and lowered serum IgM and IgG. Antibiotic treatment can also cause long-term immune alterations. Even after recolonization with microbiota from healthy donors, antibiotic-treated mice had increased frequencies of proinflammatory CCR2 + macrophages and T-bet^+^ IFN-γ^+^ T helper 1 (T_H_1)-like CD4^+^ T cells in the colon for at least 60 days after antibiotic cessation [67]. The use of broad-spectrum antibiotics led to aberrant inflammatory cytokine production in response to LPS, demonstrating an over-active gut response to microbial stimulation. Additionally, mice recolonized with bacteria after antibiotic administration had an impaired intestinal T_H_2-like response, showing decreased IL-13^+^CD4^+^ T cell frequencies during Helminth infections. Antibiotics use alters the carefully balanced relationship between the immune system and microbes, leading to dysregulated immune function and lasting impacts of virus and disease control.

Given the significant impacts that antibiotics can have on immune function, it is logical to question the role that antibiotic use might play in altering immune responses to systemic immunotherapies. Early preclinical studies found that germ free or antibiotic-treated tumor-bearing mice had worse responses to ICIs. Lida et al. found that germ-free and antibiotic-treated melanoma-bearing mice had impaired anti-tumor immune response after CpG and anti-IL10 receptor (aIL-10R) treatment, as evidenced by reduced TNF production, co-stimulatory CD86 expression, and IL-12 production in tumor-infiltrating immune cells [68]. Antibiotic-treated mice had larger tumors and shorter survival, even with aIL-10R and CpG treatment, when compared to healthy controls in multiple tumor types including lymphoma, colon cancer, and melanoma. Mechanistic experiments showed that gut microbiota activated Toll-like receptor 4 (TLR4), resulting in modulation of tumor-infiltrating immune cells toward a pro-inflammatory, TNF^+^ phenotype. *Ruminococcus* and *Alistipes* species were found to be significantly depleted by antibiotics, and administration of *Alistipes shahii* species to antibiotic-treated mice was able to restore TNF production in tumor-infiltrating myeloid cells.

In a separate study, mice bearing melanoma or sarcoma tumors and treated with broad spectrum antibiotics for 2 weeks were shown to have significantly worse survival with PD-1 and CTLA-4 blockade than mice not given antibiotics [63]. ICI responses could be rescued in germ-free mice treated with antibiotics with a FMT from ICI responding patients, but not non-responding patients. Further examination of the microbial communities of responders revealed a high enrichment of *Akkermansia muciniphila.* Oral administration of *A. muciniphila* was able to restore anti-PD-1 responses in antibiotic-treated mice, highlighting the potential of microbial-supplementation to alter ICI response.

These preclinical studies provided the rationale that the gut microbiome interaction with ICIs may influence clinical outcomes and also showed that manipulation of the microbiome through prebiotics, probiotics, or FMT may alter response rates and incidence of adverse events.

Although there has been some conflicting literature regarding the effect of recent antibiotic use on ICI efficacy in the clinic, a recent meta-analysis including all cancer type found antibiotic use to be associated with reduced overall survival (*HR* = 3.38; 95% *CI* = 2.05–2.75) and progression free survival (*HR* = 1.84; 95% *CI* = 1.49–2.26); however, patient level factors were not controlled for in this analysis[69]. Specifically in melanoma, antibiotic exposure within 3 months prior to ICI resulted in significantly worse OS (*HR* = 1.81, 95% *CI* = 1.27–2.57) on multivariable cox-proportional analysis for stage IV disease when controlled for age, sex, checkpoint class, substage, and surgical procedure [70]. Median OS was longer for those without antibiotic was significantly prolonged at 43.7 versus 27.4 months, *p* = 0.01. Further research is warranted to determine the precise impacts of antibiotic administration given the many potential confounders regarding antibiotic use on ICI response to or as a marker of general health and comorbidities.

### Microbiome and colitis

Colitis and diarrhea are one of the most common immune related adverse events with checkpoint inhibitor use [57, 71, 72]. The most common symptoms of ICI-induced colitis include diarrhea (92%), abdominal pain (82%), hematochezia (64%), fever (46%), and vomiting (36%) [73]. Anti-CTLA-4-induced colitis presents at a median time of onset 4 weeks after first infusion [74]. Conversely, onset of anti-PD-1-induced colitis ranges from 2 months up to 2 years after initial infusion [75]. A meta-analysis of randomized control trials of checkpoint inhibitors found the relative risk of diarrhea and colitis in ICI treatment of 1.64 (*p* = 0.002) versus control treatments with relative risk of high-grade diarrhea of 4.46 (*p* = 0.008) and colitis 15.81 (*p* < 0.001) [76].

The pathophysiology of ICI-mediated colitis is complex and incompletely understood. Preclinical studies showed that CTLA-4 knockout mice develop fatal enterocolitis due to T-cell proliferation [77]. Another proposed mechanism is through anti-CTLA-4 antibodies modulating the microbiota-intestinal barrier and inducing apoptosis [78]. Risk and prognostic factors for ipilimumab-induced colitis include elevated serum IL-17 level, peripheral eosinophilia, and NSAID use [73, 79, 80].

In a prospective analysis of patients to be put on anti-CTLA-4 therapy, *bacteroides* phyla were found to be enriched in patients who did not develop colitis on checkpoint inhibitors. Vetizou et al. showed that oral administration of *Bacillus fragilis* and *Bacillus cepacia* can restore anti-CTLA-4 response and reduce severity of immune-mediated colitis [42]. In a separate prospective study of 34 metastatic melanoma, patients who developed ipilimumab-associated colitis (*n* = 10) had decreased Bacteroidetes in pre-treatment fecal samples [81].

Chaput et al. also found that baseline fecal samples enriched in *Firmicutes* phylum (*Ruminococcus*, *Lachnospiracea incertae sedis*, *Blautia*, *Clostridium* IV, *Eubacterium*, unclassified *Lachnospiraceae*, and *Pseudoflavonifracto*) were more likely to develop colitis (*p* = 0.09) and higher baseline *Bacteroidetes* were less likely to develop colitis (*p* = 0.011)[60]. In this study, higher incidence immune-induced enterocolitis associated with better cancer outcome and vice versa. Patients who developed ICI-induced colitis had measurably lower serum levels IL-6, IL-8, sCD25, and regulatory T cells prior to ipilimumab administration. The proposed mechanism was through higher expression CTLA-4 on Tregs thereby causing their inhibition while consequently inducing effector T cell activation, resulting in both antitumor effects and colitis (an immune related adverse event).

One case series found that fecal microbiota transplant induced histological and clinical remission for steroid-refractory ICI-induced colitis [82]. In samples from the lamina propia, these patients had predominantly CD8^+^ T cells at the time of refractory colitis and higher CD4 FoxP3 + after FMT. This study found no association between alpha-diversity and either incidence of colitis or efficacy of FMT. Another study found higher proportion of CD8 T cells in anti-PD-1-induced colitis versus greater CD4 T cells in anti-CTLA-4-induced colitis [83].

A retrospective analysis of 327 cancer patients found that those with ICI-induced diarrhea or colitis had better overall survival compared to patients without GI symptoms [84]. Earlier studies of ipilimumab in melanoma patients also showed association between treatment-related adverse events and improved response rate [85, 86]. An investigation of 198 patients with either melanoma or renal cell carcinoma on ipilimumab found that the 39 patients who developed colitis had significantly higher tumor response rates [87].

## Microbiome and other anti-melanoma therapies

While the interplay of the microbiome with immunotherapeutics is the focus of this review as the first line and most effective treatments against metastatic melanoma, other therapeutics such as radiation, targeted therapy, and rarely systemic chemotherapy may be used as well. There is a relative dearth of information on the microbiome and its interactions with BRAF-targeted therapies or with systemic chemotherapy in melanoma.

### Radiation and the microbiome

Radiation has long been hypothesized to cross-prime anti-tumor T cells to cause tumor regression in non-target lesions through the so-called abscopal effect [88]. However, the relationship between the gut microbiome and this potential abscopal effect is newly forthcoming and not yet substantially studied in melanoma in the preclinical or clinical setting.

In a mouse model, radiotherapy (RT) administered after gut microbiome alteration through vancomycin antibiotic administration to mice was found to potentiate the RT-induced antitumor response through decrease in tumor growth. This synergy was mediated through CD8 + T cells and interferon gamma [89]. Another preclinical study by Shiao et al. showed that commensal bacteria and fungi differentially mediated tumor response to RT. Mice injected with breast tumor cells and melanoma cells which received antibiotics before administration of RT subsequently had faster tumor growth and decreased survival. After antibiotic administration, commensal gut bacteria were replaced by fungi. They concluded commensal bacteria may exert anti-tumor effects by generating activated T cells after RT, while gut fungi may produce an immunosuppressive environment via T-cell and macrophage interactions [90]. Patients and mice models in various non-melanoma cancers have been shown to alter the diversity and prevalence of species. Additionally, given diarrhea, colitis, and mucositis are frequent side effects of radiation, microbial diversity at these sites is altered following RT [91].

## Diet as prevention?

Individual diets have a great effect on microbiome composition and may have downstream effect on cancer from tumorigenesis to treatment [92]. Furthermore, certain foods and diet patterns have been shown to affect the risk of cancer development in different types of cancer (e.g., aflatoxins causing hepatocellular carcinoma, Cantonese-style salted fish causing nasopharyngeal cancer, and red meat increasing colorectal cancer risk) as well as exert a protective effect (e.g., dairy/calcium supplementation’s protective effect against colorectal cancer and the Mediterranean-type diet pattern reducing risk of multiple cancers) [93, 94]. Additionally, the metabolism of oral drugs, particularly those with a narrow therapeutic index, can be greatly affected by meals and thus their efficacy and/or side effect profile may be impacted [95].

Recently, dietary impact on melanoma tumorigenesis, treatment, and outcomes has been at the forefront of scientific enquiry. As evidence emerges about the effect of the gut microbiome on immune checkpoint inhibitor response in melanoma, efforts have increasingly turned toward lifestyle and dietary modification as a potential strategy for melanoma management.

### Red meat and processed meats

According to the World Health Organization’s (WHO) International Agency for Research on Cancer (IARC), processed meats and red meats are categorized as carcinogenic and probably carcinogenic respectively due to associations or probable association with development of stomach cancer, colorectal cancer, and prostate cancer. Certain methods of cooking and processing meat generate known carcinogenic compounds, such as polycyclic aromatic hydrocarbons, N-nitroso-compounds, or heterocyclic amines [96–99]. Results of studies on the association of red and processed meat consumption on melanoma risk have been conflicting.

In a prospective cohort of 500,000 from the National Institutes of Health-American Association for Retired Persons Diet and Health study in which improved survival was seen at the highest quartile of meat consumption (*HR*:0.82; (0.71–0.96) *p* = 0.13)); however, the trend was not statistically significant across red meat intake, suggesting this result may have been spurious [100]. More recently, in a combined analysis from two large prospective cohorts, including 75,263 women from the Nurse’s Health Study (1984–2010) and 48,523 men from the Health Professional’s Follow-up Study (1986–2010), Yen et al. found increasing red and processed meat intake to be associated with decreasing melanoma risk [101]. A dose-dependent inverse relationship was observed with increasing quintiles of intake in their samples, with *HR* 0.81 (0.70–0.95) at the highest quintile and a significant trend across increasing consumption (*p* < 0.05) after adjusting for known melanoma risk factors. The authors hypothesized the dose dependent risk relationship for melanoma may be due to potentially cancer-protective substances found in red meats, such as retinol and nicotinamide [102, 103]. However, given non-uniform results across studies, no strong association between melanoma risk and red and processed meat consumption can be inferred and the potential benefits at preventing melanoma are likely outweighed by the increased risk for other cancer types.

### Fruit, vegetable, and fiber consumption

Fibers are indigestible carbohydrates found in plant-based foods, including whole grains, fruits, and vegetables. Multiple proposed mechanisms for fiber’s anti-tumor effects include fiber’s ability to bind to bile salts that may be carcinogenic and the ability to produce short chain fatty acids when consumed, which may stop the growth of cancer cell lines [104–107]. The World Cancer Research Fund/American Institute for Cancer Research Third Expert Report on Diet, Nutrition, Physical Activity, and Cancer: Impact and Future Directions has shown fiber consumption (> 30 g per day) to be one of the strongest dietary protective effects in colorectal cancer [108–110].

A systematic review by de Waure et al. of case–control and cohort studies highlighted a pattern of reduced melanoma risk with higher intake of fruits (34–46% risk reduction with fruit consumption) and vegetable intake (40–57% risk reduction) [111]. However, one of the largest cohorts from the systematic review analyzed in their review did not find any correlation between fruit and vegetable consumption and melanoma risk and the overall protective effect was driven by smaller studies only[111–114]. A population-based case–control study by Malagoli et al. with 380 cases and matched 719 controls in Northern Italy found an inverse relationship between melanoma risk and the Dietary Approaches to Stop Hypertension (DASH) index (*OR* 0.86 95% *CI* (0.76, 0.98), *p* = 0.03) for those < 50 years, but not for the Italian Mediterranean index (IMI) when adjusted for confounders. When stratified by sex, a strengthened protective effect was seen in younger women (< 50 years) for the DASH diet and IMI, which may suggest a hormonal mechanism [115, 116]. However, they ultimately conclude that their findings also lend support to the idea that health eating patterns, including diets rich in vegetable and fruit consumption may potentially reduce melanoma risk [117].

Citrus fruits in particularly are suggested to increase melanoma risk due to naturally containing psoralens, a subgroup of furocoumarins, a compound that sensitizes the skin to UV radiation [118]. An examination of two large US cohorts (*n* = 105,432) with 24–26 years of follow-up found the risk of melanoma increased with citrus fruit consumption (≥ 1.6 times per day vs < 2 times per week with grapefruit consumption had the highest risk of melanoma development [118]. However, a recent study by Melough et al. of 388,467 Americans (3894 melanoma cases) with 15.5 years of follow-up did not find any association between citrus consumption and melanoma risk [119]. Furthermore, Sun et al. examined the association of furocoumarin intake and skin cancer risk, including melanoma risk, and found no significant association between total estimated furocoumarin intake and melanoma risk [120]. These results cast doubt on the hypothesis that increased risk or the potential mechanism by which citrus may contribute melanoma risk [112].

### Coffee and caffeine

Animal studies suggest caffeine may protect against UV-induced burn lesions in mice [121]. Lukic et al. found reduced melanoma risk in groups that consumed low to moderate amounts of filtered coffee (> 1–3 cups/day, *HR* 0.80, 95% *CI* (0.66–0.98)) and those who consumed high-moderate amounts of coffee (> 3–5 cups/day *HR* 0.77, 95% *CI* (0.61–0.97) in a large cohort of Norwegian women (*n* = 104,080) as part of the Norwegian Women and Cancer Study (NOWAC) [122]. Similarly, among 3 cohorts of healthcare professionals (*n* = 209,338) a protective effect of caffeine and noted significantly lower melanoma risk was noted in those with high caffeine intake (≥ 393 mg/day) as compared to those with lower caffeine intake (< 60 mg/day) (*HR* 0.78, 95% *CI* (0.64, 0.96) [123]. Similarly, Caini et al. also reported an inverse association between melanoma risk and caffeinated coffee consumption among 476,160 from the European Prospective Investigation into Cancer and Nutrition (EPIC) study in men (HR for highest quartile of consumption vs. non-consumers 0.31, 95% *CI* (0.14–0.69)) and not women (*HR* 0.96, 95% *CI* (0.62–1.47)) in their sample [124]. Importantly, this association was confirmed in a multiethnic cohort study from Hawaii and Los Angeles, Park et al. observed a 38% reduction in melanoma risk in non-white adults for those who consumed > 4 cups of coffee per day as opposed to no coffee (*HR* 0.72, 95% *CI* (0.52–0.99), *p* = 0.002) [125]. Two large meta-analyses by Yew et al. and Liu et al. from observational studies both confirmed these findings with a pooled relative risk for melanoma among regular coffee drinkers was 0.75 (95% *CI* (0.63–0.89)) compared with controls and the highest quantity intake vs. lowest quantity intake of 0.81 (95% *CI* (0.68–0.97), *p* = 0.003), respectively [126, 127].

### Alcohol

Alcohol consumption is thought to increase sunburn severity, acting as a photosensitizer through its metabolites, such as acetaldehyde, which consequently increases melanoma risk [128]. Its purported association with melanoma dates back to the 1977 from Third National Cancer Survey [129]. Current literature generally supports a positive association of alcohol with melanoma risk; however, the evidence is still inconsistent [112, 130]. Two large cohort studies conducted by Kubo et al. and Rivera et al. both showed a positive relationship between alcohol and melanoma risk. From the Women’s Health Initiative Observational study cohort of 59,575 women with 532 melanoma cases, white postmenopausal women who drank 7 or more drinks per week had greater melanoma risk as compared to nondrinkers over a mean follow-up period of 10.2 years *HR* 1.64 (95% *CI*: 1.09, 2.49). In addition, those who drank white wine or liquor were at more risk (*HR* 1.52, 95% *CI* (1.02, 2.27)), an association independent of sun exposure [131]. Similarly, a study Rivera et al. including 3 large US cohorts including 210,252 adults (1374 cases of melanoma) over a follow-up period of 18.3 years found greater alcohol consumption was associated with increased invasive melanoma risk (*HR* 1.14, 95% *CI* (1.00–1.29) per drink/day, *p* = 0.04). [132]. A pooled analysis of 8 case–control studies (1886 cases) and found women who ever consumed alcohol had increased risk of melanoma (*OR* = 1.3, 95% *CI* (1.1–1.5)) [133]. Meta-analysis from 14 case–control and 2 cohort studies (total of 6251 cases of melanoma) revealed any alcohol consumption was associated with increased melanoma risk as compared to no drinking/occasional drinking [134]. However, it is difficult to remove sun exposure confounding within any dietary study.

## Food and melanoma therapies

Growing evidence that a favorable microbiota may increase the efficacy of anti-PD-L1 and anti-CTLA-4 therapies by activating dendritic cell (DC) activation, leading to further anti-tumor T cell response [39, 42–44, 135]. Additional studies have re-emphasized GM importance by showing that patients with malignancy on antibiotics had significantly shorter overall survival and progression free survival on immunotherapy agents as compared to patients who were not on antibiotics [63, 136].

Given the potential to modify the GM for enhanced therapeutic response, there has understandably been greater interest in studies on dietary modifications to achieve a more favorable GM. In an analysis of dietary factors and GM composition in melanoma patients, overall diet quality and whole grain consumption were found to have a positive correlation with pro-response bacteria, indicating a favorable GM profile for improved response to immunotherapy. In contrast, increased sugar and red meat consumption was negatively correlated with the presence of pro-response bacteria [137]. Further preclinical studies showed short-chain fatty acids (SCFA), a breakdown product of dietary fiber, to increase anti-PD-1 efficacy and the prebiotics mucin and inulin were also shown to improve anti-tumor immunity to melanoma [138, 139]. High-fiber diet interventions have been shown to change microbial composition in study populations, especially increases in fiber-degrading microbes *Bifidobacterium* and *Lactobacillus* [140]. In a preclinical study by Keuhm et al., mice with B16 melanoma on a high fructose diet were resistant to immunotherapy. Cultured melanoma cells exposed to fructose in culture increased HO-1 expression driving resistance to immunotherapy, which could then be reversed in vitro with HO-1 inhibitors [141].

Studies on dietary interventions in melanoma outcomes and treatment efficacy have also focused on the possible benefit of probiotics. Probiotics refer to live organisms that when consumed in appropriate quantities may have health benefits [142]. They can be found in supplement form or in a variety of foods, including certain yogurts and kombucha [143]. Thus far, animal studies have shown some potential benefits to probiotic use in reducing cancer risk and mucosal inflammation; however, supporting human studies on probiotic use are lacking. One likely mechanism of probiotics is through modulation of the GM. In patients with cancer, modulation of the GM to be more favorable may possibly enhance therapeutic response. From animal studies, other proposed anti-tumor mechanisms of probiotics have included greater regulation of T_regs_ or TGF-β and increased recruitment of Th17 via the CCL20/chemokine receptor 6 axes in metastatic disease, both mechanisms of which involve alterations to the tumor microenvironment [144, 145].

A recent study by Spencer et al. examined dietary fiber intake and probiotic use in a large cohort of melanoma patients on immune checkpoint inhibitors (*n* = 128) and in mouse models. The authors found high-fiber intake was strongly correlated with fruit, vegetable, legume, and whole-grain intake and was associated with significantly increased PFS in their prospective cohort (not yet reached versus 13 months). Interestingly, PFS was greatest in the group that consumed a high fiber diet but was not on probiotics. The authors also performed parallel preclinical studies in mice and noted that mice on low fiber diets had worse response to anti-PD-1 therapies, experiencing shorter time to tumor outgrowth, as compared to mice on a standard fiber-rich whole grain diet. The mechanism of poorer response to anti-PD-1 therapy on a low-fiber diet was thought to be due to suppression of intratumoral IFN-γ T cell responses [146].

Given the lack of prospective supportive human studies on probiotics use and melanoma treatment efficacy, clinical trials are therefore ongoing to study probiotics use in melanoma patients on anti-PD-1 therapy. In one trial (NCT03817125), a probiotic, SER-401, will be given orally to metastatic melanoma patients on anti-PD-1 inhibitors to both determine safety and treatment efficacy and to assess for changes in the GM of participants. Another trial (NCT0367803) will also examine the effects of a probiotic, a bacterial strain, MRx0518, in addition to an anti-PD-1 in patients with non-small cell lung cancer (NSCLC), bladder cell carcinoma, renal cell carcinoma, and melanoma [147, 148].

**Fig. 3:**
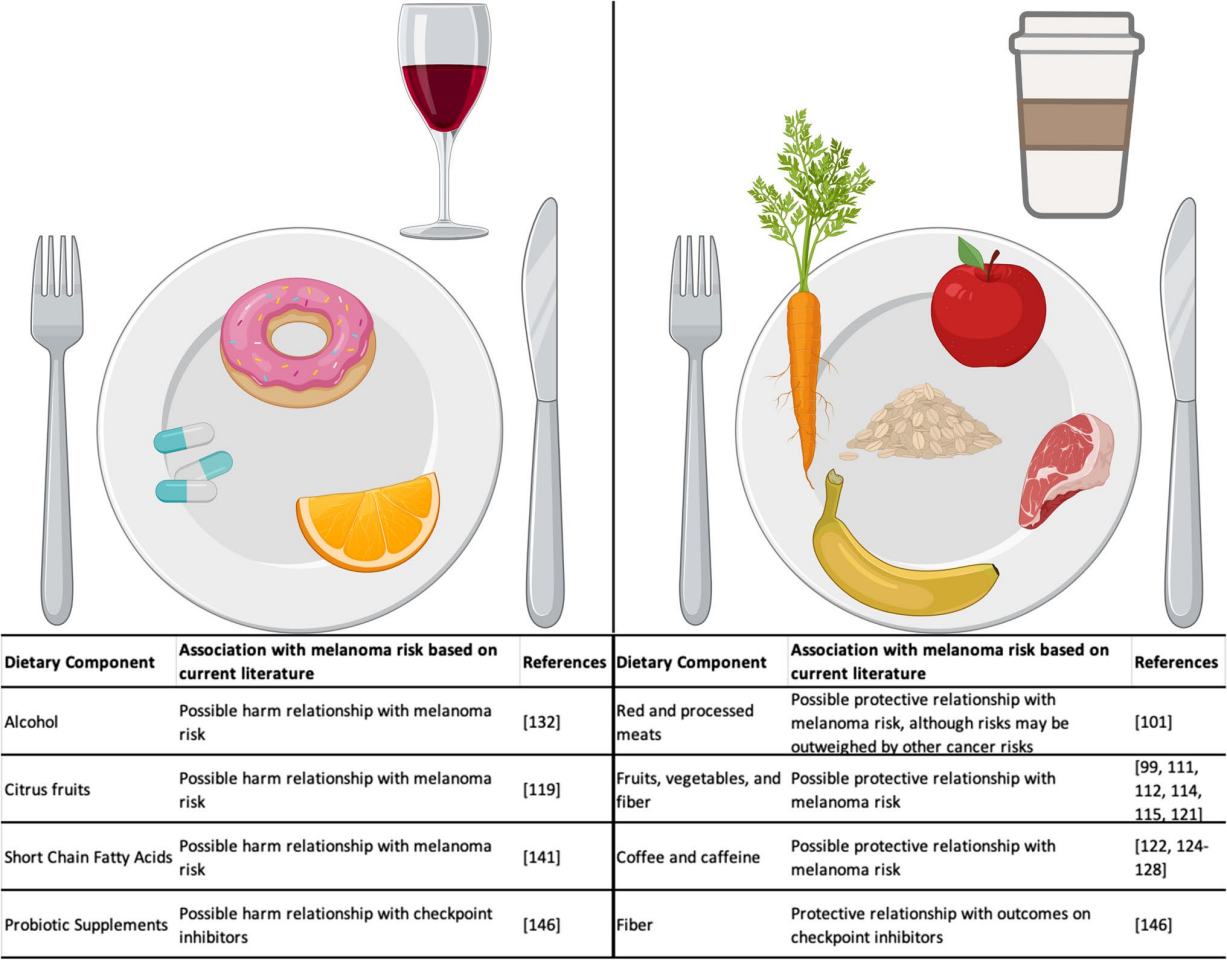
Foods associated with beneficial versus harmful effects in development or treatment of melanoma. Created with biorender.com

The allure of simple dietary modifications to affect the microbiome and its role in melanoma has naturally generated more interest in the role of dietary modification in melanoma. However, despite an abundance of studies on the association of dietary factors and melanoma, many of these studies are observational, retrospective, have smaller sample sizes, affected by recall bias, have small effect sizes, confounded by multiple factors, and have often reported conflicting results [149]. Figure 3 summarizes the foods with the strongest associations for benefits or harms. Given the lack of high-quality, randomized control studies on dietary associations and melanoma, few definitive conclusions can be drawn from the current literature. Despite the lack of high-quality evidence for dietary modifications in melanoma, it is still important to understand the potential therapeutic or carcinogenic mechanisms of dietary components in melanoma development and treatment.

## The microbiome as a therapeutic intervention

Given the significant role the microbiome has in immune presentation and the role it has taken in immunotherapy prognostication, examining the therapeutic potential is important.

### Fecal microbiota transplantation

Fecal microbiota transplantation (FMT) or stool transplantation is the procedural placement of stool of a healthy donor into another patient’s intestines. Often liquified donor stool is transferred through colonoscopy into the recipient, but other methods including feeding tube, enema, or capsules have been utilized[150]. FMT has shown success in treatment of the clostridium difficile infection and a randomized trial of the inflammatory bowel condition ulcerative colitis [151, 152].

Recently, two first-in-human clinical trials were published in *Science* by Davar et al. and Baruch et al. testing the addition of FMT to checkpoint inhibitors in checkpoint inhibitor refractory patients [153, 154]. In the first trial, Davar et al. reported a prospective enrollment of 16 patients who experienced PD-1 primary refractory disease after ≥ 2 cycles of anti-PD-1 therapy as determined by RECIST criteria. Refractory patients were given a single administration of FMT via colonoscopy along with pembrolizumab every 3 weeks. Three patients experienced partial responses and 3 additional experienced stable diseases giving an ORR of 20% and CBR of 40%. Through serial stool microbiota examinations, the gut microbiome responders shifted toward donor samples more so than non-responders. Additionally, they found alteration of the serum cytokine environment, most notably in IL-8 increase which correlated to adverse prognosis in melanoma [155]. In a second phase 1 trial reported by Baruch et al., 10 patients with PD-1 refractory melanoma at any point during therapy with checkpoint inhibitors. Patients were administered a microbiota depleting antibiotic cocktail followed by oral stool capsules and then reinduction of anti-PD-1 therapy nivolumab with oral FMT capsules for 6 combined treatment cycles every 14 days until day 90. However, it should be noted that both partial responses occurred in patients with prior complete or prior response and > 6 months without recurrence [154].

While results of the first FMTs combined with checkpoints are encouraging, caution must be given in interpreting a non-controlled non-randomized early phase trial, especially in the context that checkpoint rechallenges have often led to ORR in the 10–20% rate [156, 157]. Excitingly, two randomized trials have been registered. The first randomized trial prospective randomized clinical trial assessing the tolerance and clinical benefit of fecal transplantation in patients with melanoma treated with CTLA-4 and PD1 inhibitors (PICASSO) has begun at Hôpitaux de Paris utilizing MaaT013 in combination with PD1 and CTLA-4. MaaT013 is a product of standardized richness containing a pooled-donor, full-ecosystem intestinal microbiome of approximately 455 species administered via enema every 3 weeks following an evacuating enema [158]. In a second trial, patients with stage III or IV melanoma who have progressed on checkpoint inhibitors (NCT04577729) will be evaluated in a randomized fashion, the effect of allogeneic versus “sham” autologous FMT. The allogenic FMT donors will be obtained from former metastatic melanoma patients in remission for ≥ 1 year from checkpoint inhibitor, while the control group of autologous FMT will be given their own stool in a “sham” procedure [159].

Procedural FMT through placement of sample directly by enema or endoscopy has inherit risks and requires a patient willing to undergo this procedure, oral capsules for administration of FMT are an attractive alternative. Additional results are awaited for a 20-person phase 1 trial (NCT03772899) which adds FMT tablet administration to approved checkpoint inhibitors [160]. The FMT-LUMINATE (NCT04951583) trial in metastatic cutaneous or uveal melanoma and non-small cell lung cancer will administer FMT capsules in combination with standard of care immunotherapy [161]. NCT04521075 is another phase 1b trial of FMT capsules and checkpoint inhibitors for unresectable and metastatic melanoma [162].

### Microbial supplementation

A multicenter phase 1b trial (NCT03817125) evaluating the addition of SER-401 (a purified suspension of firmicute spores from healthy human donors formulated into capsules) versus placebo in combination with nivolumab has begun; however, slow enrollment and COVID-19 emergence led to premature termination of the trial after only 14 patients were enrolled [163, 164].

An additional randomized phase 1 trial (NCT03934827) is utilizing a neoadjuvant microbiome approach with MRx0518, a proprietary enterococcus product. Patients enrolled will be randomized to placebo versus MRx0518 capsules to be taken twice daily for 2–4 weeks prior to surgery. Primary outcome is safety and tolerability with secondary outcomes of tumor marker measurement and overall survival [165].

### Behavioral diet-directed interventions

In lieu of procedurally microbiome modification for therapeutic benefit, alternatively, it may be possible to alter the diet of a melanoma patient to change the microbiome for therapy [166]. Enrollment to The Effect of Diet and Exercise on ImmuNotherapy and the Microbiome (EDEN) trial (NCT04866810) has begun as an intervention to add behavioral modifications to checkpoint inhibitors in patients with unresectable melanoma. Up to 30 patients will randomized into each arm for control versus intervention with a plant-based, high-fiber diet with at least 150 min of moderate or 75 min of high-intensity exercise per week [167].

Ongoing and completed trials are summarized below in Table 1. Therapeutic strategies combining checkpoint with microbiome directed interventions including FMT or microbial supplements have illustrated feasibility in early phase and pilot trials. However, randomized clinical trial data is needed to prove therapeutic manipulation of the microbiome as an efficacious cancer therapy in melanoma. 

"**Your remedy is within you, but you do not sense it… You presume you are a small entity, but within you is enfolded the entire universe.**"

**-Imam Ali.**

## Conclusion

**Table:** 

•	Diet studies are marred by confounders, but a high-fiber diet, rich in vegetables and non-citrus fruits, coffee, and unprocessed foods may exert a protective effect on melanomagenesis
•	When using checkpoint inhibitors, probiotic use and antibiotic use was associated with worsened outcome, while high-fiber diet was associated with improved outcomes
•	Work is ongoing to identify key microbes that may be predictive of checkpoint response or therapeutic adjunct
•	Fecal microbiota transplant combined with checkpoint inhibitors has been shown to induce responses in checkpoint-inhibitor resistant melanomas in a pilot trial
•	Ongoing trials continue to evaluate the effect of changing the microbiome for melanoma treatments through dietary supplements, fecal microbiota transplant, or microbial supplements

From the lab to the clinic, we have just begun to unravel the complex layers of the microbiome, immune system, and their role in oncogenesis and cancer therapy. Presently, the field is dominated by a large collection of retrospective, single-institution, small, and/or uncontrolled clinical studies. Many different microbes and foods have been proposed as potential biomarkers for response or melanoma development, however many may be biomarkers of good constitution. The promise of harnessing the power of the immune system from within for cancer therapy is exciting but is too early to tell what role microbiome treatments will play. We anxiously await the completion of ongoing and future prospective controlled trials to bring the microbiome into the clinic.

## Abbreviations

CTLA-4: Cytotoxic T cell lymphocyte antigen-4
FMT: Fecal microbial transplant
GF: Germ free
GM: Gut microbiome
HO1: Hemeoxygenase 1
ICI: Immune checkpoint inhibitors
IFN: Interferon
IL: Interleukin
LAG3: Lymphocyte activation gene-3
LPS: Lipopolysaccharide
OS: Overall survival
PD-1: Programmed death receptor 1
PFS: Progression free survival
SCFA: Short chain fatty acids
RT: Radiotherapy
TGF-Beta: Tissue growth factor beta
TMP: Tail length tape measure protein
TVEC: Talimogene laherparepvec
WHO: World Health Organization
US: United States
UV: Ultraviolet
